# The flexible cotransfer of plasmids drives the dissemination of *tet*(X4) in swine *Escherichia coli*

**DOI:** 10.1186/s13567-026-01744-8

**Published:** 2026-04-24

**Authors:** Zhenxin Hao, Chaojun Zhang, Junling Cui, Shaochuan Xing, Zhengyu Wei, Hua Wu, Jianhua Liu, Xiaoyuan Ma, Zhongyi Fang, Li Yuan

**Affiliations:** 1 College of Veterinary Medicine, Henan Agricultural University, Zhengzhou, 450046 People’s Republic of China; 2 Henan Institute of Technical Research on Agricultural Product, Zhengzhou, 450046 People’s Republic of China; 3 Key Laboratory of Quality and Safety Control of Poultry Products (Zhengzhou), Ministry of Agriculture and Rural Affairs, Zhengzhou, 450046 People’s Republic of China; 4 Ministry of Education Key Laboratory for Animal Pathogens and Biosafety, Zhengzhou, 450046 People’s Republic of China

**Keywords:** *tet*(X4), tigecycline resistance, plasmid transmission, *Escherichia coli*

## Abstract

The spread of the *tet*(X4) gene threatens the efficacy of tigecycline, a last-resort antibiotic. To understand this threat, we studied its prevalence and spread in *Escherichia coli* from pigs in China. Our investigation of 190 swine *E. coli* from Henan Province identified 26 (13.68%) as *tet*(X4)-positive. Whole-genome sequencing and phylogenetic analysis revealed considerable genetic diversity across multiple sequence types. Among these, we identified a globally epidemic O11:H25-ST457 clone. Conjugation assays confirmed high transferability, with 76.92% (20/26) of *tet*(X4)-harboring plasmids being successfully transferred, predominantly through independent transfer or cotransfer with other plasmids. We further elucidated, for the first time, the molecular mechanism of an IS*15DI*-mediated fusion between a *tet*(X4)-bearing IncHI1A/IncHI1B/IncFIA plasmid and an IncFIC(FII)/IncR plasmid. However, the resulting fusion plasmid was highly unstable without antimicrobial selection. Additionally, we report a *tet*(X4)-positive IncHI2/IncHI2A plasmid whose flexible multidrug resistance region readily underwent rearrangements during conjugation, generating heterogeneous plasmids in transconjugants. Our findings demonstrate that *tet*(X4) dissemination is primarily driven by the flexible cotransfer of plasmids rather than stable fusion events, highlighting an ongoing and dynamic threat of tigecycline resistance dissemination from livestock to the global health landscape.

**Supplementary Information:**

The online version contains supplementary material available at 10.1186/s13567-026-01744-8.

## Introduction

The spread of antibiotic-resistant bacteria poses a serious threat to global public health by significantly increasing morbidity and mortality and has attracted considerable attention. The rapid rise of multidrug-resistant (MDR) gram-negative bacteria, particularly carbapenemase-producing or tigecycline-resistant *Enterobacteriaceae*, has severely compromised the effectiveness of antibiotics [1, 2]. Tigecycline is considered a last-resort antibiotic for the treatment of severe infections caused by extensively drug-resistant bacteria. Although tigecycline has not been approved for clinical use in animals in China, an increasing number of studies have reported a rising prevalence of tigecycline-resistant isolates in animals [3, 4]. The emergence of plasmid-mediated tigecycline resistance genes, such as *tet*(X) variants and *tmexCD-toprJ*, has facilitated the global dissemination of tigecycline resistance [5, 6]. Among these, *tet*(X4) is the most prevalent variant, conferring high-level resistance to tigecycline [7].

The *tet*(X) variants encode flavin-dependent monooxygenases and represent a novel mechanism of tigecycline resistance [8]. Numerous studies have shown that mobile elements such as IS*CR2* [3] and conjugative plasmids (e.g., IncQ, IncX1, IncFIB, and IncHI1) [9, 10] play a critical role in the horizontal dissemination of *tet*(X4) in *Enterobacteriaceae*. The *tet*(X4) gene can undergo rolling-circle transposition, leading to changes in the orientation or replacement of surrounding insertion sequences (e.g., IS*26*, IS*1*), thereby generating flexible and distinct genetic contexts [11]. Furthermore, our research group discovered that a *tet*(X)-positive plasmid, pT28R-1, could achieve horizontal transfer by fusing with a conjugative helper plasmid [12], which can further enhance the flexibility of *tet*(X) in spreading across diverse bacterial hosts.

Since the discovery of *tet*(X4), numerous studies have investigated the prevalence of tigecycline resistance across different regions of China, revealing both regional and source-specific variations in its prevalence as well as in the risk of horizontal transmission [11, 13, 14]. However, in Henan Province, one of the major pig-producing regions in China, data on the prevalence of *tet*(X4)-positive swine *E. coli* are limited, and the dynamics of their horizontal transmission remain poorly understood. To address this gap, we collected 190 *E. coli* isolates from pig farms in Henan Province during 2023–2024. Among these, 26 (13.68%) were identified as *tet*(X4)-positive, and we further analyzed the mechanisms underlying the horizontal transmission of the resistance gene.

## Materials and methods

### Bacterial isolates

During 2023–2024, a total of 190 nonreplicate *E. coli* isolates were initially identified from 300 anal swabs collected from pig farms in Henan Province, China. Species identification was subsequently confirmed using the VITEK-2 Compact system (Biomerieux, Marcy l’Etoile, France) [15] and 16S rRNA gene sequencing.

### Antimicrobial susceptibility testing (AST)

The broth microdilution method was employed to determine the minimum inhibitory concentrations (MICs) of eight antimicrobials in accordance with the guidelines of the Clinical and Laboratory Standards Institute (CLSI, 2020) and the European Committee on Antimicrobial Susceptibility Testing [16, 17]. The antimicrobial agents tested were tigecycline, doxycycline, imipenem, ceftiofur, amikacin, colistin, florfenicol, and enrofloxacin. *E. coli* ATCC25922 served as a quality control strain. Three independent biological replicates were performed.

### Whole-genome sequencing (WGS) and bioinformatic analysis

The total genomic DNA of tigecycline-resistant *E. coli* was extracted individually using the E.Z.N.A^®^Bacteria DNA kit (OMEGA) and subjected to WGS using the Illumina HiSeq2500 platform (Illumina, San Diego, CA, USA). Draft genomes were assembled from raw Illumina short reads using SPAdes (version 4.2.0) with predefined parameters. Multilocus sequence typing (MLST) was determined via the online tool [18] on the basis of the draft assemblies. Serotypes, plasmid replicon types, and antimicrobial resistance genes (ARGs) were identified using SerotypeFinder (version 2.0), PlasmidFinder (version 2.1), and ResFinder (version 4.6) on the CGE server [19], respectively. Insertion sequence (IS) elements were determined using ISfinder (version 2.1) [20]. Contigs from the draft assemblies were annotated using Prokka (version 1.12) for downstream analysis. Following annotation, phylogenetic analysis of the annotated contigs was conducted using Roary and FastTree, leveraging single-nucleotide polymorphism (SNP) distances. The resulting phylogenetic trees were visualized using iTOL (version 5) [21].

To further explore the horizontal dissemination mechanism of *tet*(X4), we selected representative strains ZH142 and ZH177 and their transconjugants T142A and T177A for WGS using Illumina Novaseq6000 and Oxford Nanopore Technologies (ONT) MinION platforms. Thereafter, the resulting short- and long-read data were assembled using Unicycler (version 0.4.8) with a hybrid strategy. The whole sequence was subsequently annotated using the RAST server [22] and manually corrected. Then, serotypes, plasmid replicon types, ARGs, and IS elements were identified according to the methods mentioned above. The comparative analysis and plasmid maps were performed using BRIG and Easyfig [15, 23].

### Conjugation and S1-pulsed-field gel electrophoresis (S1-PFGE)

The conjugation assays were conducted utilizing the *tet*(X4)-positive isolates as the donors and *E. coli* J53 (resistance to sodium azide) as the recipient according to our previous study, with some modification [24, 25]. Considering that the *tet*(X4)-carrying IncHI1 plasmid is a thermo-sensitive plasmid, the donor and the recipient mated in a 4:1 ratio at 28 °C and 37 °C for 12 h, respectively. The transconjugant was screened on MacConkey agar supplemented with tigecycline (2 µg/mL) and sodium azide (200 µg/mL). The presence of *tet*(X4) in the transconjugants was confirmed with polymerase chain reaction (PCR). Plasmid profiles of the donors and their transconjugants were further verified using S1-PFGE [26]. All mating assays were replicated at least three times biologically.

### Biological features of the fusion plasmid

To explore whether the fusion plasmid pT142A imposes any additional fitness costs on its host cell T142A, we determined the growth curves of the isolate ZH142 and its transconjugants, including T142A (carrying pT142A), T142B (carrying pZH142-1), and T142C (carrying pZH142-1 and pZH142-2), following established methods [27, 28]. Subsequently, mating assays were performed with strains T142A and T142B as donors and *E. coli* C600 as the recipient using the method described above. The conjugation efficiency was then calculated.

We then assessed the stability of the fusion plasmid pT142A following a previously described protocol [29]. Briefly, transconjugants T142A, T142B, and T142C were cultured in Luria–Bertani (LB) broth without antibiotics and subcultured daily at a 1:100 ratio into fresh medium for 25 days. Every 48 h, bacterial cultures were serially diluted in 0.9% saline and plated onto nonselective and tigecycline-containing LB agar plates. All plasmid stability assays were replicated three times biologically. Additionally, the presence of the *tet*(X4) and *tet*(A) genes, as well as the fusion junctions in plasmid pT142A were verified by PCR using the primers listed in Table 1.

**Table 1:** **Primer sequences used in this study**

Primer name	Primer sequence (5′–3′)	Size of product (base pairs [bp])
*tet*(X4)-F	CTGATTCGTGTGACATCATCTTTTG	204
*tet*(X4)-R	GTTAAATTTCCCATTGGTCAGATTA	
FS1-F*	GGACACAAAAACGGTTGGGG	5575
FS1-R*	AACAGTGCCGGTTGACGAAG	
FS2-F*	TCGCCTTTCACGTAGTGGAC	2803
FS2-R	GCGGGGCTAACAGGTAAGAT	
*tet*(A)	CCTCCTGCGCGATCTGGTTC	627
*tet*(A)	TCCTCGCCGAAAATGACCCAA	

Primer sequences used to verify fusion sites are denoted with an asterisk (^*^)

#### S1-PFGE of the transconjugant T177A

After overnight incubation of the transconjugant T177A, the culture broth was plated onto LB agar. Following this, ten colonies were randomly selected, individually inoculated into fresh LB broth, and grown to an optical density at 600 nm (OD_600_, optical density at 600nm) of 0.5. The plasmid profiles of these cultures were confirmed by S1-PFGE.

## Results

### Antimicrobial resistance and multidrug-resistant profiles of swine *E. coli* isolates

**Figure 1:**
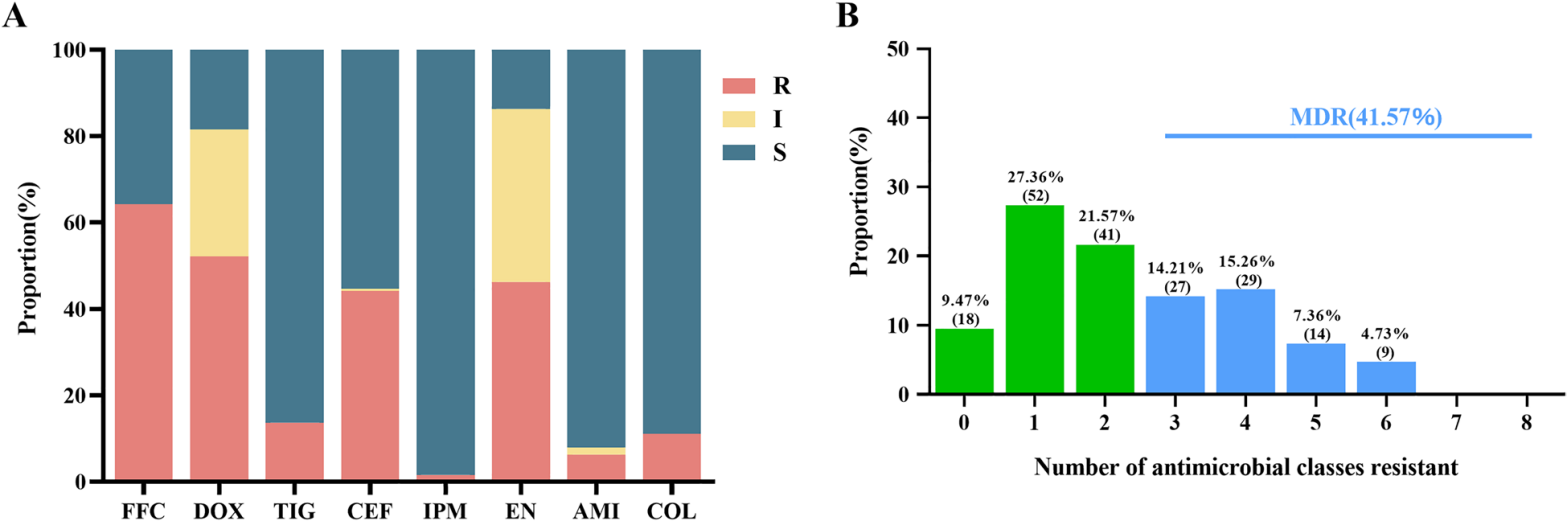
**Resistance phenotypes of 190 *E. coli* from pigs in Henan Province, China**. **A** Antibiotic resistance rates. **B** Multidrug resistance rates. The numbers in parentheses indicate the quantity of the isolates. R resistance, I intermediate, S susceptibility, FFC florfenicol, DOX doxycycline, TIG tigecycline, CEF ceftiofur, IPM imipenem, EN enrofloxacin, AMI amikacin, COL colistin, MDR multidrug-resistant.

To determine the antimicrobial resistance profile, all 190 *E. coli* isolates were subjected to antimicrobial susceptibility testing. As shown in Figure 1A, the isolates displayed high rates of resistance to florfenicol (122/190, 64.21%), doxycycline (99/190, 52.10%), enrofloxacin (88/190, 46.31%) and ceftiofur (84/190, 44.21%). The detailed MIC information for all 190 isolates is provided in Additional file 1. In contrast, lower resistance rates were observed against tigecycline (26/190, 13.68%), imipenem (3/190, 1.57%), colistin (21/190, 11.05%), and amikacin (12/190, 6.31%). The notably low resistance to tigecycline and imipenem is likely attributable to their prohibition for clinical use in animals in China. Conversely, the moderate resistance to colistin and amikacin, despite their approved use, may reflect the ongoing selection pressure from their application. Furthermore, 41.57% (79/190) of the isolates were classified as multidrug-resistant (MDR) (Figure 1B).

### Unveiling the genomic epidemiology of *tet*(X4)-positive *E. coli* in swine

**Figure 2:**
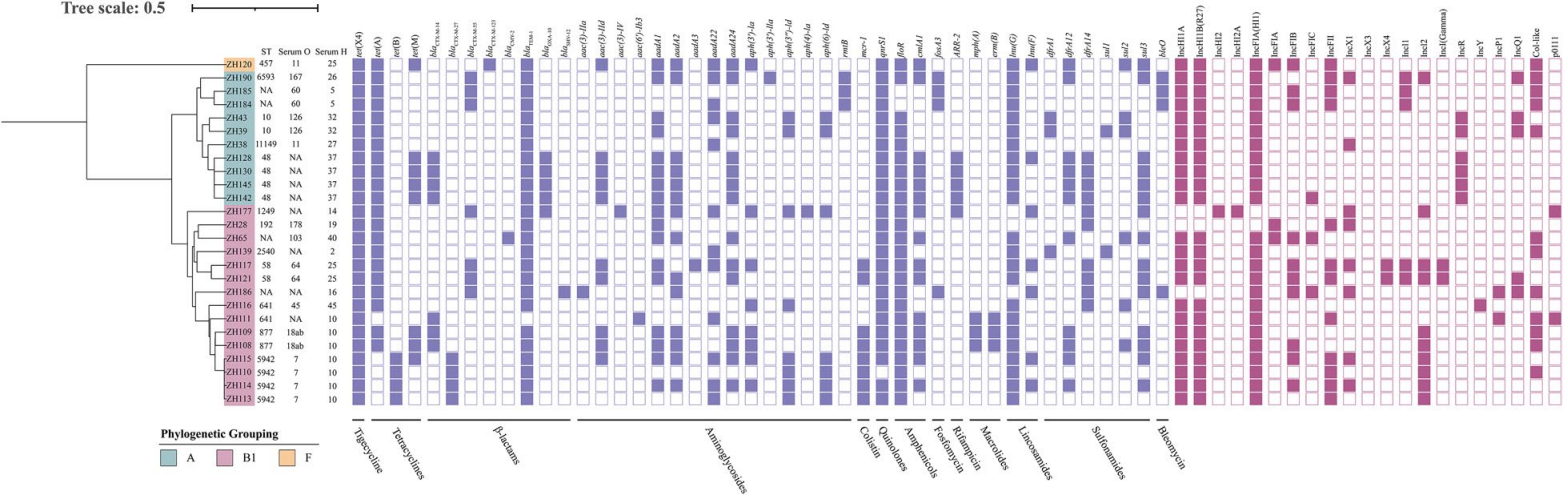
**Phylogenetic analysis of the core genomes of 26 *tet*(X4)-positive *E. coli* isolates**. Colored regions represent the phylogenetic groups of the tested isolates. Antimicrobial resistance genes and plasmid types are shown in blue and red, respectively. Isolates for which MLST or serotype could not be determined were denoted as not assigned (NA) in the phylogenetic tree.

The genomic characteristics of the 26 tigecycline-resistant isolates were analyzed on the basis of Illumina whole-genome sequencing (WGS) and are summarized in Figure 2. As expected, all of the isolates harbored *tet*(X4), suggesting that the acquisition of *tet*(X4) was the primary driving factor for the development of tigecycline resistance in porcine *E. coli* from Henan Province. Multilocus sequence typing (MLST) analysis revealed that the 26 isolates were assigned to 12 known sequence types (STs) and 4 non-typable STs, indicating considerable genetic diversity, and suggesting that* tet*(X4) was likely acquired through horizontal gene transfer.

Phylogenetic grouping of the 26 *tet*(X4)-positive *E. coli* isolates showed that the majority belonged to group B1 (15/26, 57.69%), followed by group A (10/26, 38.46%). A single isolate, ZH120, was identified as group F. Further characterization revealed that ZH120 was a globally disseminated O11:H25-ST457 epidemic clone, which had been reported in both humans and animals [30]. Single-nucleotide polymorphism (SNP) analysis revealed that, apart from the isolate ZH120 clustering into a single lineage, 96.2% (25/26) of the *tet*(X4)-positive *E. coli* was classified into another clade, which was further subdivided into two sub-branches. A significant genetic divergence (SNP > 15 000) was observed between the distinct clades, indicating considerable genetic diversity among the *tet*(X4)-positive *E. coli* from pigs, which was consistent with the abovementioned MLST results. The most prevalent plasmid replicon types were IncHI1A, IncHI1B(R27), and IncFIA(HI1), collectively identified in 88.46% (23/26) of the isolates. This high prevalence suggests that these are the primary plasmid vectors responsible for harboring and disseminating the *tet*(X4) gene. Analysis of antimicrobial resistance gene (ARG) profiles demonstrated that all *tet*(X4)-positive *E. coli* isolates co-carried genes conferring resistance to β-lactams and aminoglycosides. Furthermore, a high prevalence of ARGs against other drug classes was observed, including those for quinolones (25/26), lincosamides (25/26), tetracyclines (25/26), amphenicols (24/26), and sulfonamides (20/26). Further analysis showed that the *tet*(X4) gene was frequently co-located with other ARGs, most notably *bla*_TEM-1_ (25/26), *qnrS1* (25/26), *floR* (24/26), *lnu*(G) (23/26), and *tet*(A) (21/26). The concurrent presence of these multiple resistance genes suggests that nearly all *tet*(X4)-positive *E. coli* are MDR, a scenario that significantly complicates clinical infection control.

### Horizontal transfer traits of the *tet*(X4)-bearing plasmid

**Figure 3:**
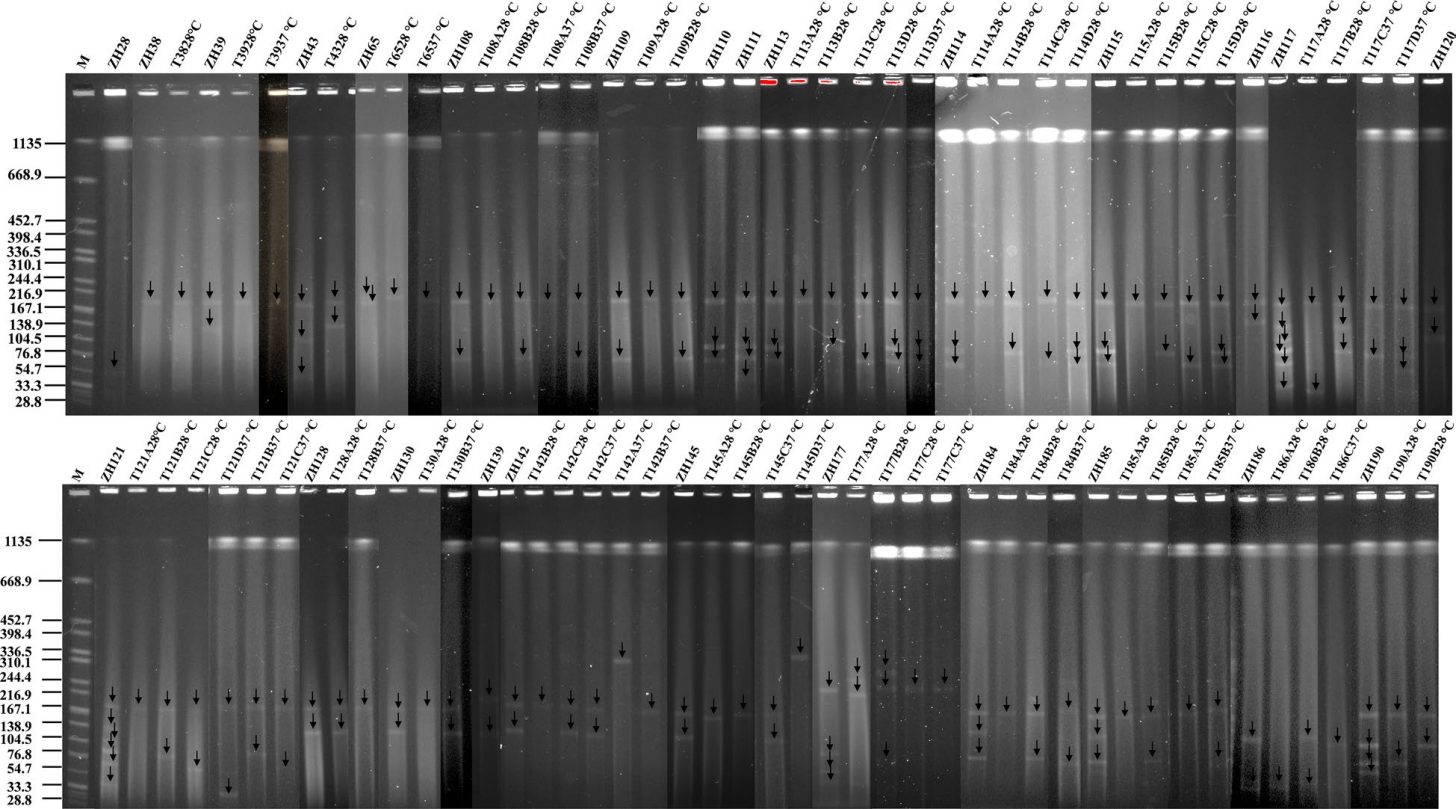
**S1-PFGE profiles of transconjugants and their parental strains.** All strains with names starting with the letter “T” represent transconjugants. Letters **A**–**D** represent transconjugants carrying different numbers of plasmids. Mating assays were conducted at 28 °C and 37 °C. “M” denotes the molecular weight marker, *Salmonella enterica* serotype Braenderup H9812. The recipient strain is *E. coli* J53.

Conjugation assays demonstrated that the *tet*(X4)-bearing plasmid from 20 out of 26 donor *E. coli* isolates (76.92%) could be successfully transferred into the recipient *E. coli* J53 at 28 °C. Among these, plasmids from 15 donors (15/20, 75%) were also transferable at 37 °C (Figure 3). A total of 51 distinct transconjugants were obtained from these 20 donors, among which 21 transconjugants carried a single plasmid, and 30 contained at least two plasmids. This result indicates that the *tet*(X4)-bearing plasmids can undergo horizontal transfer efficiently at both temperatures, either independently or cotransferred with other plasmids. With the exception of isolates ZH28 and ZH38, 24 out of the 26 *tet*(X4)-positive isolates (92.30%) harbored at least two plasmids, indicating a high potential for plasmid fusion during conjugation (Figure 3). However, fusion events were observed in only two transconjugants, T142A^37 °C^ and T145D^37 °C^ (2/51, 3.92%), in which the *tet*(X4)-bearing plasmids were significantly larger than those in their respective donors (ZH142 and ZH145). On the basis of the increased plasmid size and conserved resistance profiles, we inferred that recombination events likely occurred between the *tet*(X4)-bearing plasmid and other plasmids during the conjugation process. Collectively, these results demonstrate that *tet*(X4)-bearing plasmids in *E. coli* are far more likely to undergo cotransfer with other plasmids than to fuse with them prior to transfer.

### WGS analysis of the strain ZH142 and transconjugant T142A

WGS revealed that the donor isolate ZH142 (ST48) contained a chromosome of 4 618 319 bp and two resistance plasmids, pZH142-1 (201 203 bp) and pZH142-2 (143 847 bp). In the transconjugant T142A, a single fusion plasmid, pT142A (345 888 bp), was identified. The size of pT142A was approximately equal to the sum of pZH142-1 and pZH142-2 (Table 2), indicating that it likely originated from the fusion of these two parental plasmids. The chromosome of T142A was 4 633 100 bp in length (Table 2).

**Table 2:** **Chromosome and plasmid genetic characteristics**

Strains/plasmids	Size (bp)	Plasmid types	Resistance genes
ZH142	4,618,319	Chromosome	*aadA1*, *bla*_OXA-10_, *cmlA1*, *floR*, *qnrS1*, *ARR-2*, *dfrA14*, *tet*(A)^*^
pZH142-1	201,203	IncHI1A/IncHIB(R27)/ IncFIA(HI1)	*aac(3)-IId*, *aadA22*, *bla*_TEM-1b_, *bla*_CTX-M-14_, *lnu(G)*, *qnrS1*, *floR*,*tet*(X4)
pZH142-2	143,847	IncFIC(FII)/IncR	*aadA2*, *aadA1*, *cmlA1*, *sul3*, *tet*(M), *tet*(A), *dfrA12*
T142A	4,633,100	Chromosome	None
pT142A	345,888	IncHI1A/IncHIB(R27)/ /IncFIA(HI1)/ IncFIC(FII)/IncR	*aac(3)-IId*, *aadA22*, *bla*_TEM-1b_, *bla*_CTX-M-14_, *lnu(G)*, *qnrS1*, *floR*,*tet*(X4), *aadA2*, *aadA1*, *cmlA1*, *sul3*, *tet*(M), *tet*(A), *dfrA12*
ZH177	4,910,626	Chromosome	None
pZH177-1	254,204	IncHI2/IncHI2A	*aph(4)-Ia*, *aph(6)-Id*, *aadA22*, aadA1^*^, *aadA2b*, *aph(3′)-Ia*, *aph(3″)-Ib*, *aac(3)-IV*, *lnu(F)*, *bla*_OXA-10_, *bla*_TEM-1b_, cmlA1^*^, *floR*, *qnrS1*, *ARR-2*, *sul3*, *tet*(A)^*^, *tet*(X4), *dfrA14*
pZH177-2	99,263	p0111	None
pZH177-3	62,492	IncI2	None
pZH177-4	46,712	IncX1	*aph(3′)-Ia*, *bla*_CTX-M-55_, *qnrS1*, *tet*(A)
pZH177-5	4065	None	None
pZH177-6	1623	None	None
T177A	4633,121	Chromosome	None
pT177A-1	265,142	IncHI2/IncHI2A	*aph(4)-Ia*, *aph(6)-Id*, *aadA22*, aadA1^*^, *aadA2b*, *aph(3′)-Ia*, *aph(3″)-Ib*, *aac(3)-IV*, *lnu(F)*, *bla*_OXA-10_, *bla*_TEM-1b_, cmlA1^*^, *floR*, qnrS1^*^, *ARR-2*, *sul3*, *tet*(A)^*^, *tet*(X4), *dfrA14*
pT177A-2	232,447	IncHI2/IncHI2A	*aph(4)-Ia*, *aph(6)-Id*, *aadA22*, *aadA1*, *aadA2b*, *aph(3′)-Ia*, *aph(3″)-Ib*, *aac(3)-IV*, *lnu(F)*, *cmlA1*, *floR*, *sul3*, *tet*(A)^*^, *tet*(X4)
pT177A-3	46,712	IncX1	*aph(3′)-Ia*, *bla*_CTX-M-55_, *qnrS1*, *tet*(A)

^*^≥ two copies

**Figure 4:**
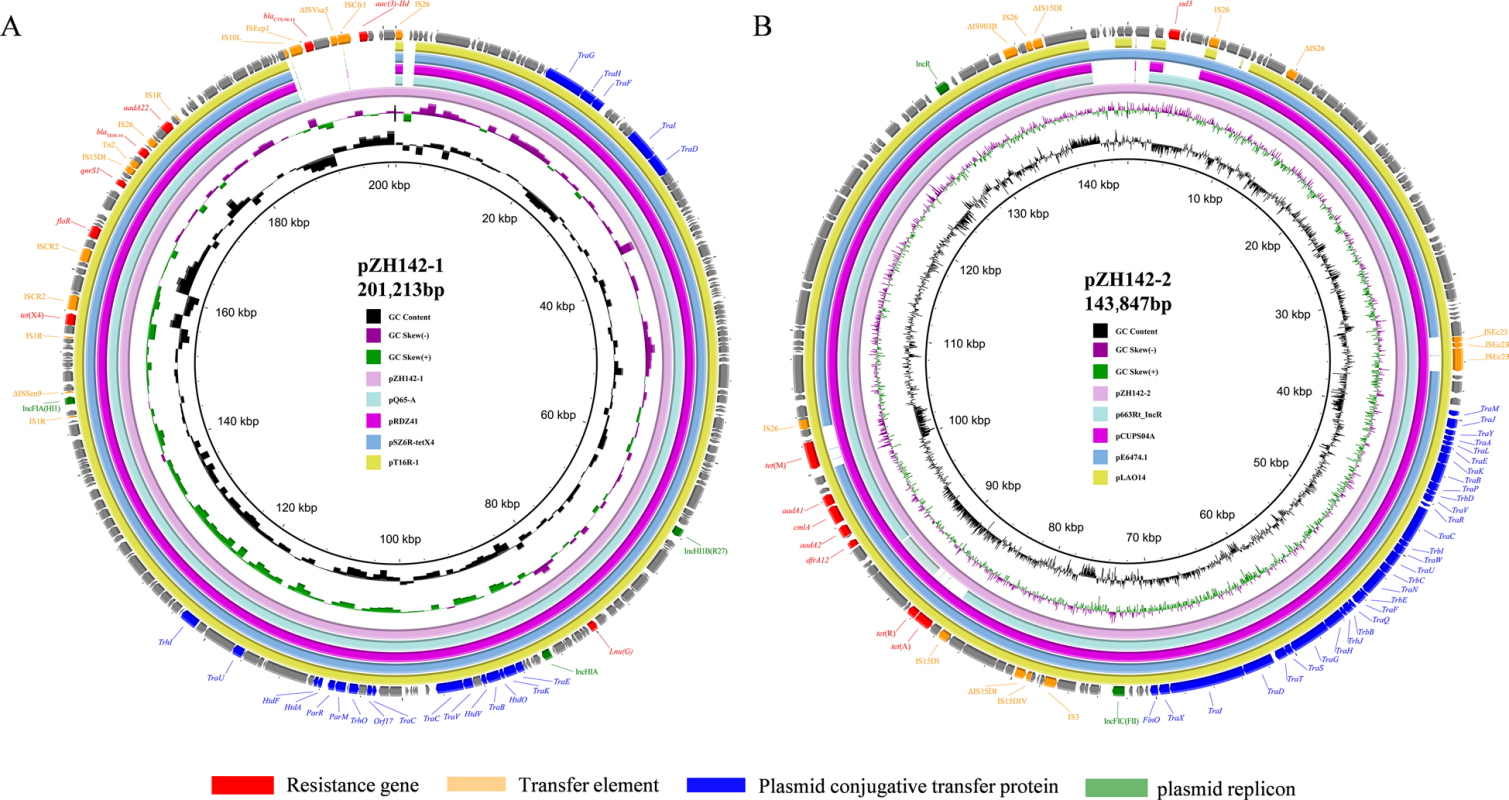
**Circular alignments of plasmids pZH142-1** **A** **and pZH142-2** **B** **with related plasmids.** The outer ring represents the annotated features of the reference plasmid. Gaps indicate regions that are absent or divergent relative to the reference plasmid.

The plasmid pZH142-1 was identified as an IncHI1A/IncHIB(R27)/IncFIA(HI1) type with an average guanine and cytosine (GC) content of 46.60%. It harbored multiple resistance genes, including *tet*(X4), *bla*_CTX-M-14_, *bla*_TEM-1b_, *qnrS1*, *floR*, *aadA22*, *aac(3)-IId*, and *lnu(G)* (Table 2). As shown in Figure 4A, pZH142-1 exhibited high genetic identity (99.66–99.75%) and 94% coverage with several known plasmids from diverse sources in China, such as pQ65-A (CP180223, sputum, *E. coli*, 2023), pT16R-1 (CP046717, pet dog, *E. coli*, 2019), pSZ6R-tetX4 (MW940627, pork, *Citrobacter rodentium*, 2019), and pRDZ41 (CP139495, human, *Klebsiella pneumoniae*, 2020). This broad distribution across hosts and recent years highlights the wide dissemination and broad host range of this IncHI1A/IncHIB(R27)/IncFIA(HI1) plasmid backbone in China. Strikingly, pZH142-1 contained an additional multidrug resistance region (MRR) carrying *bla*_CTX-M-14_ and *aac(3)-IId*, along with multiple insertion sequences (ISs), which was not present in the other compared plasmids. These findings suggest that the IncHI1A/IncHIB(R27)/IncFIA(HI1) plasmid can continuously diversify its resistance profile by acquiring mobile genetic elements.

The plasmid pZH142-2 was identified as an IncFIC(FII)/IncR type with an average GC content of 51.96%. It carried multiple resistance genes, including *aadA2*, *aadA1*, *cmlA1*, *sul3*, *tet*(M), *tet*(A), and *dfrA12* (Table 2). Comparative analysis showed that the backbone sequence of pZH142-2 was highly similar to those of several known IncFIC(FII)/IncR plasmids from humans and animals in Asia (Figure 4B), such as pLAO14 (OP242228, *Homo sapiens*, *E. coli*, Laos), p663Rt_IncR94 (CP080076, *Homo sapiens*, *E. coli*, China, 2019), pCUPS04A (CP180695, slaughterhouse, *E. coli*, Thailand, 2020), and pE6474.1 (CP116982, *Sus scrofa*, *E. coli*, Thailand, 2021). A key distinction, however, was that pZH142-2 harbored a greater number of resistance genes.

**Figure 5:**
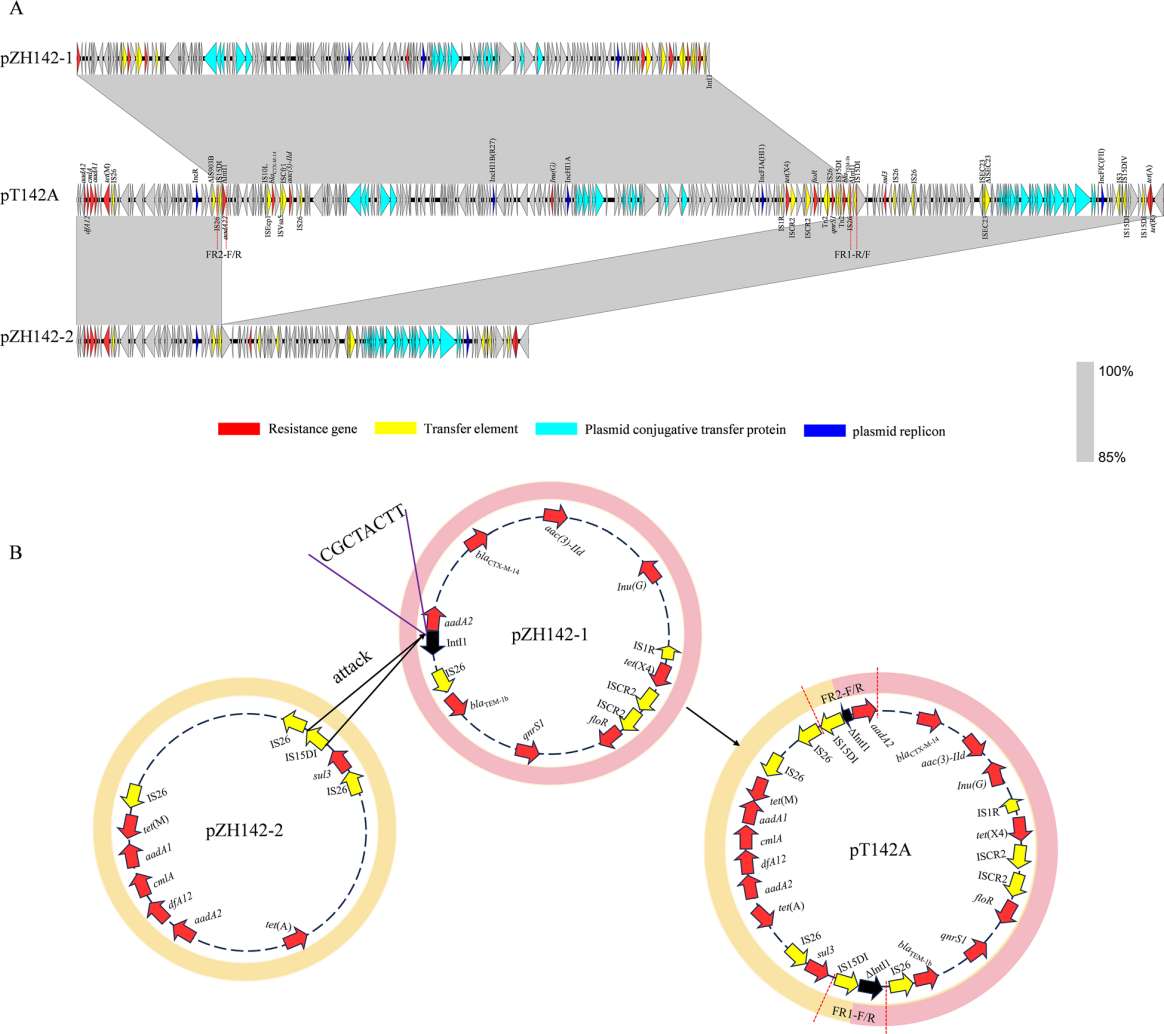
**Mechanism of fusion plasmid formation.** As indicated in the figure legend, different colors represent genes with different functions. The dotted part is the fusion-site primer sequence. **A** Comparative analysis of the linear sequences of the parental plasmid and the fusion plasmid. The shaded area indicates the homologous sequences shared between the plasmids. **B** The proposed model for the formation of the fusion of plasmids. The fusion plasmid pT142A was formed by IS*15DI*-mediated recombination between pZH142-1 and pZH142-2, resulting in truncation of IntI1 and generation of an 8-bp target site duplication (CGCTACTT).

The fusion plasmid pT142A had an average GC content of 48.84% and carried all the resistance genes from both pZH142-1 and pZH142-2 (Table 2; Figure 5A). A proposed model for its formation is shown in Figure 5B. Specifically, an IS*15DI* element located upstream of the *sul3* gene on pZH142-2 likely targeted the site “CGCTACTT” within the integrase gene (*intI1)* on pZH142-1, resulting in the truncation of *intI1* and the linearization of pZH142-1. The linearized pZH142-1 was then integrated into pZH142-2, forming the fusion plasmid pT142A. This recombination process generated an 8-bp target site duplication (TSD; CGCTACTT) and introduced an additional copy of IS*15DI* upstream of the integrated pZH142-2 segment. Structurally, the fusion plasmid pT142A is composed of sequences from pZH142-1 (1–46 096 nucleotides [nt] and 248 138–345 888 nt) and pZH142-2 (46 097–247 309 nt). An 828-bp segment (247 310–248 137 nt) at the junction contains an IS*15DI* copy and an 8-bp repetitive sequence (CGCTACTT).

### Biological characteristics of the fusion plasmid pT142A

**Figure 6:**
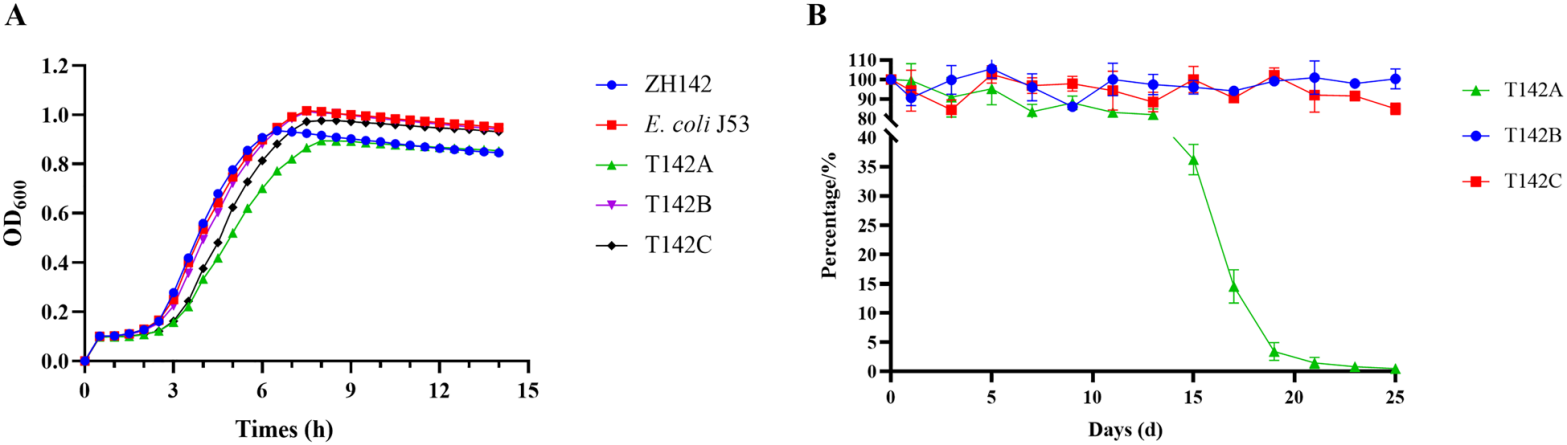
**Fitness cost associated with three types of ZH142 transconjugants.** **A** Growth curves of the parental isolate ZH142 and its transconjugants. **B** Stability of plasmids pT142A and pZH142-1 in the corresponding host strains. T142A (harboring the fusion plasmid pT142A), T142B (harboring pZH142-1), and T142C (harboring both pZH142-1 and pZH142-2).

Growth kinetics of five strains ZH142, T142A, T142B, T142C and *E. coli* J53 are shown in Figure 6A. No significant growth difference was observed among these strains, indicating that the fusion plasmid pT142A did not impose a detectable fitness cost on its bacterial host. As expected, the conjugation frequency of pZH142-1 at 37 °C decreased by approximately 10^4^-fold to (3.75 ± 1.63) × 10^–8^, compared with (5.30 ± 1.48) × 10^–4^ at 28 °C, demonstrating that its transfer was remarkably temperature-sensitive (Table 3). In contrast, the conjugation frequencies of the fusion plasmid pT142A were (2.49 ± 0.70) × 10^–5^ at 37 °C and (1.41 ± 0.08) × 10^–5^ at 28 °C, which were within a similar range. This result suggests that the horizontal transfer of pT142A has lost its sensitivity to temperature.

**Table 3:** **Conjugation frequencies of T142A and T142B at different temperatures**

Parental strains	Plasmids	Recipient strains	Temperature (℃)	Transfer rates
T142A	pT142A	*E. coil* C600	28	(1.41 ± 0.08) × 10^–5^
			37	(2.49 ± 0.70) × 10^–5^
T142B	pZH142-1	*E. coil* C600	28	(5.30 ± 1.48) × 10^–4^
			37	(3.75 ± 1.63) × 10^–8^

To assess the stability of the fusion plasmid pT142A, the transconjugants T142A, T142B, and T142C were serially passaged in antibiotic-free medium for 25 days (Figure 6B). Compared with pZH142-1, the fusion plasmid pT142A was not stably maintained. A gradual loss of pT142A was observed from the outset of the experiment, resulting in a total loss of 18% by day 13. Subsequently, the rate of plasmid loss increased sharply. The retention rate dropped to around 36.22% by day 15 and eventually to only 0.48% by the end of the experiment. These results underscore that the fusion plasmid pT142A is inherently unstable and difficult to maintain in the absence of antimicrobial selection pressure.

### WGS analysis of the *tet*(X4*)*-positive strain ZH177 and its transconjugant T177A

**Figure 7:**
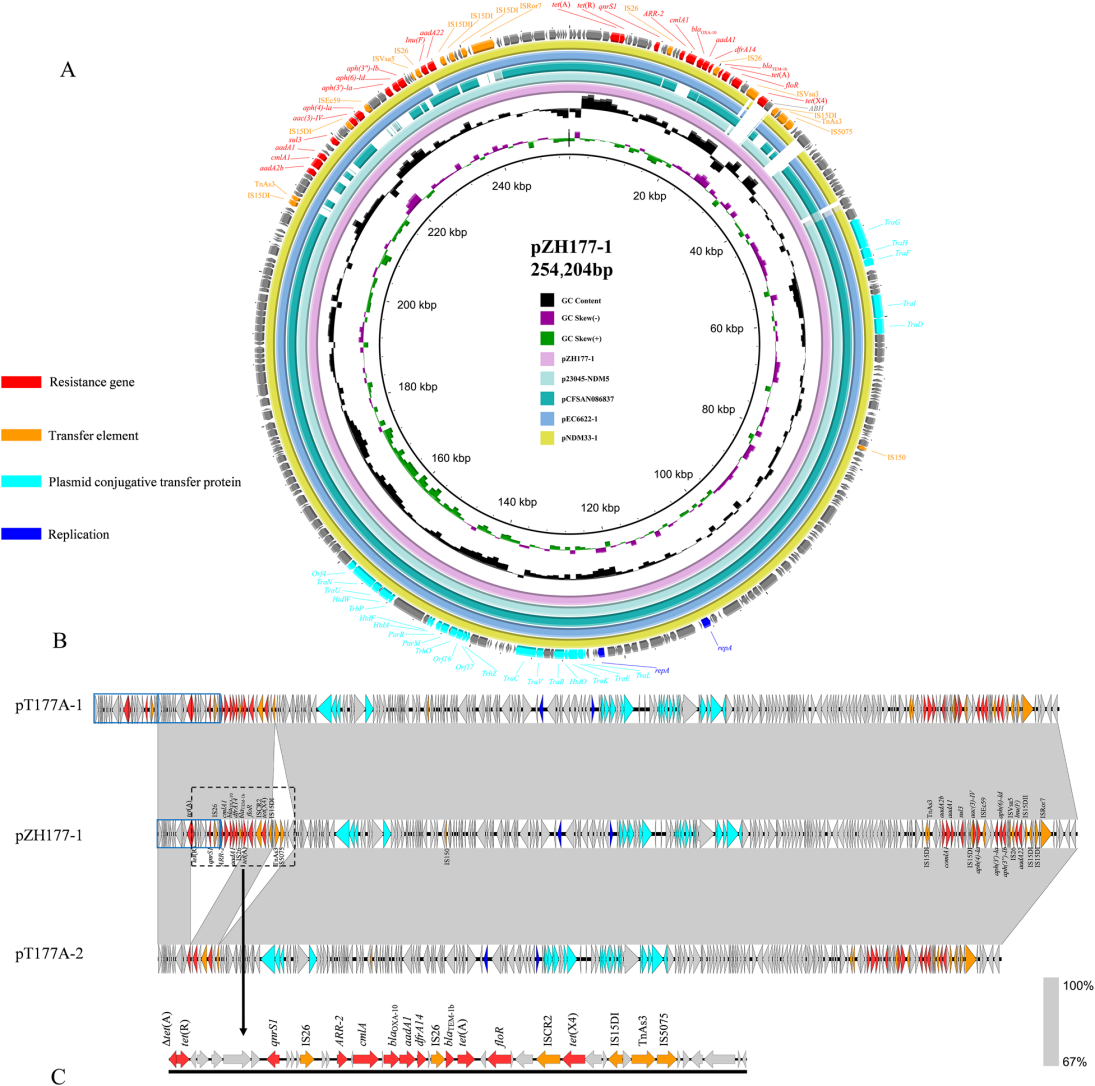
**Comparative analysis of the *tet*(X4)-positive plasmid carried by isolate ZH177 and its transconjugants**. **A** Circular genetic maps of plasmid pZH177-1 from isolate ZH177 and related plasmids deposited in the GenBank database. **B** Linear comparative analysis of plasmid pZH177-1 with plasmids pT177A-1 and pT177A-2 from transconjugant T177A. **C** Variable region of plasmid pZH177-1. Shaded regions denote homologous areas shared among plasmids. The light-blue box in panel **B** represents the repeated tandem amplification sequences in pT177A-1, and the dashed black box represents the main variable regions during the conjugation process of pZH177-1.

S1-PFGE analysis revealed that in the transconjugants of isolate ZH177, several *tet*(X4)-bearing plasmids were different from all of the original plasmids in ZH177. To investigate the underlying genetic mechanism, we performed WGS on both strain ZH177 and its transconjugant T177A (Table 2; Figure 7B). The results showed that *E. coli* ZH177 (ST1249, phylogroup B1) contained a 4 910 626-bp chromosome and six plasmids. These included two resistance plasmids: the *tet*(X4)-positive IncHI2/IncHI2A plasmid pZH177-1 (254 204 bp) and an IncX1 plasmid pZH177-4 (46 712 bp), as well as four nonresistance plasmids: pZH177-2 (99 263 bp), pZH177-3 (62 492 bp), pZH177-5 (4065 bp), and pZH177-6 (1023 bp). Plasmid pZH177-1 (GC content 47%) contained two distinct MRRs. One (named left MRR) carried 9 resistance genes, including *tet*(X4), *tet*(A), *qnrS1*, *floR*, *aadA1*, *bla*_OXA-10_, *bla*_TEM-1b_, *dfrA14*, and *ARR-2*, while the other (named right MRR) harbored 11 resistance genes, including *cmlA1*, *aph(4)-Ia*, *aph(3′)-Ia*, *aph(3″)-Ib*, *aac(3)-IV*, *aph(6)-Id*, *aadA22*, *aadA2b*, *aadA1*, *sul3*, and *lnu(F)*. The pZH177-4 (GC content 47%) carried the resistance genes *aph(3′)-Ia*, *bla*_CTX-M-55_, *qnrS1*, and *tet*(A).

To investigate the global prevalence of IncHI2/IncHI2A plasmids harboring the *tet*(X4) gene, we compared pZH177-1 with available sequences in the NCBI database. Comparative analysis revealed that pZH177-1 shared high genetic identity (99.96–100.00%) with several characterized IncHI2/IncHI2A plasmids from diverse sources (Figure 7A), including p23045-NDM5 (OR497833, *Salmonella* from human, China, 2023), pCFSAN086837 (CP039438, *Salmonella* from chicken, Vietnam, 2017), pEC6622-1 (CP096588, *E. coli* from human, China, 2021), and pNDM33-1 (CP076648, *E. coli* from duck, China, 2017). A key distinguishing feature of pZH177-1 was the presence of an IS*CR2-tet*(X4)-*ABH* module, which was absent in the other compared plasmids.

The transconjugant T177A harbored three plasmids, pT177A-1 (265 142 bp), pT177A-2 (232 447 bp), and pT177A-3 (46 712 bp) (Table 2). Compared with the donor strain ZH177, pT177A-3 was identical to pZH177-4, while both pT177A-1 and pT177A-2 were highly similar to pZH177-1, except for structural variations in the left MRR (Figure 7B). Further sequence alignment indicated that a 17 207-bp tandem duplication comprising a *tet*(A)-*tet*(R)-*qnrS1*-IS*26* module was present upstream of the left MRR in pT177A-1. Concurrently, a 6239-bp fragment containing Tn*As3* and IS*5075* was deleted downstream of this region. In pT177A-2, the left MRR underwent more extensive deletion. It lost not only the 6239-bp downstream fragment, but also a 15 518-bp upstream segment that encompassed *tet*(R)-*qnrS1*-IS*26*-*ARR-2*-*cmlA1*-*bla*_OXA-10_-*aadA1*-IS*26*-*bla*_TEM-1b_-*tet*(A). Consequently, the MRR in pT177A-2 was streamlined and contained only the *tet*(A), *floR*, and *tet*(X4) resistance genes.

**Figure 8:**
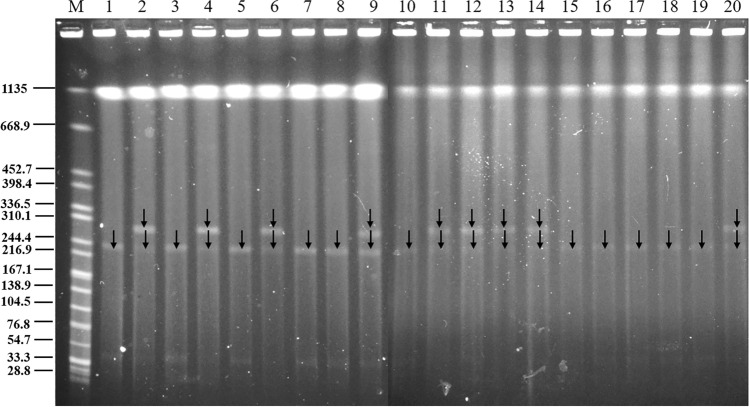
**S1-PFGE analysis of transconjugant T177A.** A total of 20 single colonies of transconjugant T177A were randomly selected for S1-PFGE analysis. One transconjugant carried a large plasmid, whereas another exhibited heterogeneity, with some colonies harboring a large plasmid and others carrying an even larger plasmid.

Given the incompatibility of plasmids in one cell, we hypothesized that during the transfer process of pZH177-1, the left MRR (Figure 7C) was prone to rearrangement. This resulted in a heterogeneous population of transconjugants, wherein some subpopulations carried pT177A-1, while others carried pT177A-2. To test this, 20 colonies were randomly selected from an overnight culture of T177A on the LB agar plate and were subjected to S1-PFGE analysis (Figure 8). As expected, 11 out of the 20 colonies (55%) carried pT177A-2 but not pT177A-1, confirming our hypothesis. However, despite repeated attempts, we were unable to successfully isolate any colonies that carried pT177A-1 in the absence of pT177A-2, indicating that pT177A-1 might be structurally unstable and difficult to maintain as a discrete element.

## Discussion

In the present study, we isolated a swine-origin *E. coli* strain, ZH120, which carried *tet*(X4) and was identified as serotype O11:H25, sequence type ST457, and phylogenetic group F. The ST457 lineage was first reported in 2008 from a human urinary tract infection in the UK [31]. Since then, it has been detected in diverse samples of human and animal sources around the world [32–34], indicating that ST457 is a broad-host-range and globally disseminated *E. coli* clone. Furthermore, increasing evidence demonstrates that the O11:H25-ST457 clone can cause urinary tract [31], bloodstream [35, 36], and abdominal [37] infections in humans. Its close genetic relationship between human and food-producing animal isolates [30, 38] underscores its potential as a zoonotic pathogen.

To date, *E. coli* ST457 has been documented to carry numerous critical resistance genes, including *bla*_KPC_ [39], *bla*_NDM_ [40], *bla*_CTX-M_ [41], and *mcr* [42], thereby facilitating the global dissemination of these determinants. However, reports of ST457 harboring the *tet*(X) gene and conferring tigecycline resistance remain scarce. In our study, we identified one strain, *E. coli* ZH120, that co-carried *tet*(X4) and *bla*_CTX-M-123_ from 190 nonreplicate swine *E. coli*, indicating the emergence of *tet*(X4)-positive ST457 *E. coli* on pig farms in China, although tigecycline is not approved for use in veterinary medicine. Given that ZH120 not only belongs to a prevalent clone lineage but also carries *tet*(X4), we suggest that it has the potential to exacerbate the global spread of this resistance gene and pose a serious threat to clinical treatment and public health.

In recent years, there has been a growing number of reports on plasmid fusion events. These events can expand the host range of plasmids and allow them to accumulate additional resistance and virulence genes [12]. Documented examples include the fusion of an IncX1-type plasmid with an IncI1-type plasmid [43], an IS*26*-mediated fusion between an IncN1/IncF33- plasmid and an *mcr-1*-carrying phage-like plasmid [44], and the fusion of a *tet*(X4)-bearing IncHI1 plasmid with either IncF18 or IncF16 plasmids [12]. In this study, only 2 out of 51 *tet*(X4)-bearing transconjugants were found to harbor a fusion plasmid. To our knowledge, this study reports, for the first time, an IS*15DI*-mediated fusion between two conjugative resistance plasmids—IncHI1A/IncHIB(R27)/IncFIA(HI1) plasmid pZH142-1 and IncFIC(FII)/IncR plasmid pZH142-2. IncHI1-type plasmids are known to exhibit temperature-sensitive conjugation, with higher transfer frequencies at 28 ℃ and lower at 37 °C, which was consistent with the transfer characteristics of pZH142-1. Moreover, previous studies have reported that such temperature sensitivity may be lost after plasmid fusion [12]. Our observations also confirmed that the fusion plasmid pT142A displayed similar transfer frequencies at 28 °C and 37 °C, indicating that its temperature-dependent transfer ability has been lost. However, unlike the stable fusion plasmids described in previous reports [12], pT142A exhibited intrinsic instability and was difficult to maintain in the absence of antimicrobial selection pressure. This instability may be attributable to replicon interference, incompatibility between partitioning systems, or an increased metabolic burden imposed by recombination events, although the precise molecular mechanisms underlying this instability remain to be clarified. In conclusion, *tet*(X4)-bearing plasmids in swine *E. coli* are more likely to be transferred either independently or via cotransfer with other plasmids than to undergo fusion with them during the conjugation process, despite their flexible and diverse modes of horizontal transmission.

In addition, we report a swine *E. coli* ZH177, harboring a *tet*(X4)-positive IncHI2/IncHI2A plasmid. The IncHI2/IncHI2A type is a globally disseminated conjugative plasmid [45], known to carry carbapenemase genes (e.g., *bla*_NDM-5_ [46], *bla*_NDM-1_ [47]) and colistin resistance genes (e.g., *mcr-1* [48] and *mcr-9* [49]). This capacity for acquiring diverse resistance genes is facilitated by the considerable structural flexibility of its MRR. We observed that during the transfer process, the MRR frequently undergoes recombination and deletion events, resulting in heterogeneous populations of transconjugants.

## Conclusions

We systematically investigated the prevalence and horizontal transmission characteristics of *tet*(X4)-positive porcine *E. coli* from Henan Province, China. We demonstrated, for the first time, the IS*15DI*-mediated fusion of two conjugative resistance plasmids. We also identified a *tet*(X4)-positive IncHI2/IncHI2A plasmid whose MRR was highly flexible and variable, leading to heterogeneous transconjugants after conjugation. Furthermore, we discovered a clinically significant epidemic clone, *E. coli* O11:H25-ST457, coharboring *tet*(X4) and *bla*_CTX-M-123_ on a pig farm. The emergence of such strains poses a growing threat to global public health by facilitating the dissemination of tigecycline resistance.

### Nucleotide sequence accession number

The 26 *tet*(X4)-positive *E. coli* isolates were deposited under NCBI BioSample accession nos. SAMN47582671 and SAMN47582673, whereas strains ZH142, T142A, ZH177, and T177A were assigned BioSample accession nos. SAMN47582670, SAMN50554454, SAMN47582672, and SAMN50554709, respectively. Detailed information is provided in Additional files 2 and 3.

## Supplementary Information

**Additional file 1.** **Antimicrobial susceptibility results (MICs, µg/mL) for the 190 isolates included in this study.**

**Additional file 2.** **Genomic features of completely assembled chromosomes and plasmids obtained in this study.**

**Additional file 3.** **Genome assembly characteristics of 24 tigecycline-resistant Escherichia coli isolates sequenced using Illumina technology.**

## Data Availability

The 26 *tet*(X4)-positive *E. coli* isolates were deposited under NCBI BioSample accession nos. SAMN47582671 and SAMN47582673, whereas strains ZH142, T142A, ZH177, and T177A were assigned BioSample accession nos. SAMN47582670, SAMN50554454, SAMN47582672, and SAMN50554709, respectively. Detailed information is provided in Additional files 2 and 3.
