# Supplementary material for: The flexible cotransfer of plasmids drives the dissemination of tet(X4) in swine Escherichia coli
Source: Vet Res. 2026 Apr 24;57:88. doi: 10.1186/s13567-026-01744-8 (PMC13214465; doi:10.1186/s13567-026-01744-8)
Supplement: Supplementary file 1 — Additional file 1. Antimicrobial susceptibility results (MICs, µg/mL) for the 190 isolates included in this study. [file 13567_2026_1744_MOESM1_ESM.docx]

Additional file 1 Antimicrobial susceptibility results (MICs, µg/mL) for the 190 isolates included in this study.

| Strains | FFC | DOX | CEF | EN | IPM | COL | AML | TIG |
| --- | --- | --- | --- | --- | --- | --- | --- | --- |
| ZH1 | 4 | 8 | 64 | 1 | 0.06 | 0.25 | ≥512 | 0.25 |
| ZH2 | 4 | 8 | 32 | 1 | 0.25 | 0.06 | 1 | 0.5 |
| ZH3 | 16 | 8 | ≥512 | 4 | 0.13 | 0.25 | 1 | 0.25 |
| ZH4 | 2 | 1 | ≥512 | 1 | 0.06 | 1 | ≥512 | 0.25 |
| ZH5 | 4 | 8 | 32 | 2 | 0.25 | 0.03 | 1 | 0.13 |
| ZH6 | 2 | 16 | 0.25 | 0.06 | 0.03 | 0.25 | 0.5 | 0.13 |
| ZH7 | 128 | 2 | 64 | 1 | 0.06 | 0.03 | 1 | 0.5 |
| ZH8 | 4 | 8 | 32 | 8 | 0.25 | 8 | 0.5 | 0.25 |
| ZH9 | 64 | 8 | 0.25 | 2 | 0.13 | 0.13 | 1 | 0.25 |
| ZH10 | 4 | 1 | 16 | 256 | 0.03 | 0.25 | 0.5 | 0.25 |
| ZH11 | 128 | 16 | 0.25 | 1 | 0.25 | 1 | 0.5 | 0.25 |
| ZH12 | 64 | 8 | 0.5 | 1 | 0.03 | 0.06 | 0.5 | 0.06 |
| ZH13 | 64 | 4 | 0.25 | 1 | 0.13 | 0.25 | 1 | 0.13 |
| ZH14 | 2 | 8 | 0.5 | 0.5 | 0.06 | 0.5 | 0.5 | 0.25 |
| ZH15 | 128 | 8 | 0.25 | 4 | 0.06 | 0.25 | 1 | 0.5 |
| ZH16 | 4 | 32 | 16 | 1 | 0.06 | 0.25 | 0.5 | 0.25 |
| ZH17 | 4 | 8 | 64 | 1 | 0.25 | 0.25 | ≥512 | 0.5 |
| ZH18 | 4 | 8 | 64 | 1 | 0.06 | 0.13 | ≥512 | 0.25 |
| ZH19 | 4 | 2 | 256 | 256 | 0.06 | 0.5 | ≥512 | 0.13 |
| ZH20 | 2 | 16 | 0.25 | 0.5 | 0.13 | 0.5 | 1 | 0.13 |
| ZH21 | 256 | 8 | 0.5 | 2 | 0.13 | 0.13 | 1 | 0.25 |
| ZH22 | 2 | 8 | 32 | 1 | 0.25 | 0.25 | 1 | 0.25 |
| ZH23 | 128 | 16 | 8 | 1 | 0.06 | 1 | 1 | 0.25 |
| ZH24 | 256 | 16 | 0.5 | 1 | 0.06 | 1 | 1 | 0.25 |
| ZH25 | 64 | 8 | 16 | 1 | 0.06 | 0.25 | 1 | 0.25 |
| ZH26 | 2 | 2 | 16 | 0.13 | 0.13 | 0.5 | 1 | 0.25 |
| ZH27 | 2 | 2 | 0.5 | 0.5 | 0.25 | 0.25 | 1 | 0.25 |
| ZH28 | 128 | 32 | 0.5 | 1 | 0.06 | 0.5 | 1 | 8 |
| ZH29 | 128 | 8 | 0.13 | 0.5 | 0.13 | 0.25 | 1 | 0.5 |
| ZH30 | 4 | 32 | 0.25 | 2 | 0.13 | 1 | 1 | 0.25 |
| ZH31 | 128 | 8 | 16 | 2 | 0.13 | 1 | 1 | 0.25 |
| ZH32 | 4 | 8 | 1 | 2 | 0.25 | 0.5 | 1 | 0.5 |
| ZH33 | 128 | 2 | 32 | 1 | 0.25 | 0.5 | 2 | 0.25 |
| ZH34 | 4 | 8 | 0.25 | 0.06 | 0.06 | 0.25 | 1 | 1 |
| ZH35 | 64 | 8 | 16 | 1 | 0.06 | 0.25 | 1 | 0.25 |
| ZH36 | 128 | 2 | 32 | 2 | 0.13 | 0.5 | 1 | 0.25 |
| ZH37 | 128 | 2 | 16 | 2 | 0.06 | 0.5 | 1 | 0.25 |
| ZH38 | 32 | 32 | 1 | 2 | 0.13 | 0.13 | 1 | 8 |
| ZH39 | 64 | 16 | 0.5 | 1 | 0.25 | 0.25 | 1 | 8 |
| ZH40 | 1 | 0.5 | 0.5 | 0.01 | 0.06 | 0.13 | 0.5 | 0.13 |
| ZH41 | 64 | 8 | 0.5 | 1 | 0.13 | 0.25 | 1 | 0.25 |
| ZH42 | 256 | 8 | 0.5 | 4 | 0.06 | 0.5 | 1 | 0.25 |
| ZH43 | 256 | 32 | 0.25 | 1 | 0.03 | 0.5 | 1 | 8 |
| ZH44 | 2 | 16 | 0.5 | 0.03 | 0.06 | 1 | 0.5 | 0.25 |
| ZH45 | 1 | 8 | 0.5 | 0.5 | 0.13 | 0.25 | 1 | 0.25 |
| ZH46 | 2 | 32 | 0.5 | 0.06 | 0.13 | 0.5 | 1 | 0.13 |
| ZH47 | 2 | 8 | 4 | 1 | 0.5 | 0.5 | 2 | 0.25 |
| ZH48 | 64 | 16 | 32 | 0.25 | 8 | 0.13 | 2 | 0.25 |
| ZH49 | 64 | 8 | 32 | 1 | 8 | 0.25 | 1 | 0.25 |
| ZH50 | 2 | 0.5 | 1 | 0.06 | 0.25 | 1 | 1 | 0.25 |
| ZH51 | 128 | 8 | 0.5 | 2 | 0.06 | 0.25 | 1 | 0.5 |
| ZH52 | 2 | 16 | 0.25 | 0.06 | 0.13 | 0.5 | 1 | 1 |
| ZH53 | 128 | 8 | 0.25 | 1 | 0.25 | 1 | 1 | 0.25 |
| ZH54 | 4 | 0.5 | 0.5 | 0.5 | 0.06 | 0.5 | 1 | 0.25 |
| ZH55 | 2 | 1 | 0.25 | 0.25 | 0.13 | 0.25 | 1 | 0.25 |
| ZH56 | 64 | 16 | 0.5 | 0.06 | 0.06 | 0.25 | 1 | 0.25 |
| ZH57 | 2 | 32 | 0.25 | 0.03 | 0.13 | 0.5 | 1 | 0.25 |
| ZH58 | 2 | 16 | 0.5 | 0.03 | 0.02 | 0.13 | 1 | 0.25 |
| ZH59 | 2 | 1 | 0.5 | 1 | 0.13 | 0.5 | 1 | 0.25 |
| ZH60 | 2 | 0.5 | 0.25 | 16 | 0.13 | 0.25 | 1 | 0.25 |
| ZH61 | 128 | 4 | 0.5 | 0.03 | 0.25 | 1 | 1 | 0.25 |
| ZH62 | 4 | 8 | 0.5 | 1 | 0.06 | 0.5 | 0.5 | 0.25 |
| ZH63 | 4 | 32 | 0.5 | 0.06 | 0.03 | 0.25 | 0.5 | 0.13 |
| ZH64 | 4 | 8 | 1 | 0.25 | 0.13 | 0.13 | 1 | 0.25 |
| ZH65 | 64 | 32 | 16 | 32 | 0.06 | 0.13 | 1 | 8 |
| ZH66 | 128 | 32 | 0.25 | 4 | 0.25 | 0.5 | 1 | 0.25 |
| ZH67 | 64 | 8 | ≥512 | 32 | 0.13 | 0.25 | 1 | 0.5 |
| ZH68 | 64 | 4 | 1 | 1 | 0.06 | 0.25 | 1 | 0.25 |
| ZH69 | 64 | 8 | 0.25 | 1 | 0.13 | 0.25 | 0.5 | 0.25 |
| ZH70 | 64 | 8 | 0.5 | 1 | 0.03 | 0.25 | 0.5 | 0.25 |
| ZH71 | 1 | 16 | ≥512 | 0.5 | 0.03 | 0.25 | 1 | 0.25 |
| ZH72 | 128 | 8 | 0.5 | 1 | 0.06 | 0.25 | 1 | 0.25 |
| ZH73 | 2 | 1 | 1 | 1 | 0.03 | 0.25 | 1 | 0.13 |
| ZH74 | 128 | 32 | 0.5 | 0.13 | 0.03 | 0.5 | 0.5 | 1 |
| ZH75 | 1 | 8 | 0.13 | 8 | 0.03 | 0.13 | 0.5 | 0.13 |
| ZH76 | 1 | 4 | 0.25 | 16 | 0.02 | 0.03 | 0.5 | 0.13 |
| ZH77 | 2 | 32 | 0.25 | 1 | 0.13 | 0.25 | 1 | 0.25 |
| ZH78 | 2 | 4 | 0.5 | 32 | 0.06 | 0.06 | 1 | 0.25 |
| ZH79 | 32 | 4 | 0.25 | 0.5 | 0.03 | 0.13 | 0.5 | 0.25 |
| ZH80 | 1 | 0.5 | 0.5 | 16 | 0.06 | 0.25 | 1 | 0.25 |
| ZH81 | 0.5 | 4 | 1 | 32 | 0.03 | 0.25 | 0.5 | 0.13 |
| ZH82 | 64 | 8 | 0.5 | 1 | 0.03 | 0.5 | 1 | 0.25 |
| ZH83 | 64 | 8 | 0.5 | 1 | 0.03 | 0.25 | 0.5 | 0.25 |
| ZH84 | 4 | 8 | 0.5 | 1 | 0.03 | 0.25 | 1 | 0.25 |
| ZH85 | 64 | 16 | 0.5 | 256 | 0.03 | 0.25 | 0.5 | 0.25 |
| ZH86 | 64 | 16 | 256 | 2 | 0.03 | 0.25 | 1 | 0.25 |
| ZH87 | 2 | 16 | 0.25 | 0.25 | 0.06 | 0.25 | 0.5 | 0.25 |
| ZH88 | 128 | 8 | 1 | 32 | 0.06 | 0.13 | 1 | 0.25 |
| ZH89 | 2 | 8 | 0.5 | 256 | 0.03 | 0.13 | 1 | 0.25 |
| ZH90 | 64 | 16 | 0.5 | 4 | 0.03 | 0.25 | 1 | 0.5 |
| ZH91 | 128 | 16 | 0.25 | 0.5 | 0.03 | 0.25 | 1 | 0.25 |
| ZH92 | 64 | 4 | 0.25 | 0.25 | 0.06 | 0.06 | 1 | 0.25 |
| ZH93 | 2 | 8 | 0.5 | 0.5 | 0.13 | 0.13 | 0.5 | 0.25 |
| ZH94 | 64 | 8 | 0.5 | 0.5 | 0.25 | 0.13 | 0.5 | 0.25 |
| ZH95 | 64 | 8 | 0.5 | 0.5 | 0.25 | 0.13 | 0.5 | 0.25 |
| ZH96 | 2 | 2 | 0.25 | 0.06 | 0.06 | 0.13 | 1 | 0.13 |
| ZH97 | 128 | 16 | ≥512 | 2 | 0.5 | 0.06 | 0.5 | 0.13 |
| ZH98 | 128 | 8 | ≥512 | 2 | 0.13 | 0.13 | 1 | 0.25 |
| ZH99 | 128 | 64 | 128 | 2 | 16 | 0.25 | 0.5 | 0.25 |
| ZH100 | 4 | 4 | 0.5 | 1 | 0.25 | 0.5 | 0.5 | 0.25 |
| ZH101 | 128 | 16 | 1 | 0.5 | 0.13 | 0.25 | 1 | 0.25 |
| ZH102 | 64 | 16 | 0.25 | 0.25 | 0.13 | 0.13 | 1 | 0.25 |
| ZH103 | 2 | 32 | 0.5 | 0.03 | 0.13 | 0.06 | 1 | 0.25 |
| ZH104 | 256 | 4 | 512 | 1 | 0.06 | 0.5 | 2 | 0.25 |
| ZH105 | 2 | 32 | 0.5 | 16 | 0.06 | 0.5 | 1 | 0.25 |
| ZH106 | 64 | 8 | ≥512 | 1 | 0.13 | 0.13 | 0.5 | 0.25 |
| ZH107 | 128 | 4 | 512 | 2 | 0.02 | 0.25 | 1 | 0.25 |
| ZH108 | 64 | 32 | 128 | 16 | 0.06 | 8 | 1 | 4 |
| ZH109 | 64 | 32 | 128 | 16 | 0.13 | 8 | 1 | 4 |
| ZH110 | 32 | 32 | 512 | 32 | 0.13 | 8 | 1 | 8 |
| ZH111 | 64 | 32 | 512 | 32 | 0.06 | 0.25 | 4 | 8 |
| ZH112 | 64 | 32 | 512 | 32 | 0.13 | 8 | 1 | 0.25 |
| ZH113 | 64 | 32 | ≥512 | 64 | 0.06 | 8 | 1 | 8 |
| ZH114 | 64 | 64 | ≥512 | 64 | 0.06 | 8 | 1 | 8 |
| ZH115 | 256 | 64 | ≥512 | 64 | 0.03 | 8 | 1 | 4 |
| ZH116 | 64 | 32 | 0.5 | 1 | 0.06 | 0.5 | 1 | 8 |
| ZH117 | 32 | 32 | ≥512 | 8 | 0.13 | 8 | 0.25 | 8 |
| ZH118 | 4 | 32 | ≥512 | 256 | 0.25 | 8 | 1 | 0.13 |
| ZH119 | 4 | 32 | 256 | 256 | 0.06 | 16 | 0.25 | 0.25 |
| ZH120 | 128 | 32 | ≥512 | 64 | 0.06 | 0.13 | 1 | 4 |
| ZH121 | 32 | 32 | ≥512 | 8 | 0.06 | 8 | 0.25 | 4 |
| ZH122 | 64 | 8 | ≥512 | 128 | 0.25 | 16 | ≥512 | 0.03 |
| ZH123 | 32 | 16 | ≥512 | 128 | 0.13 | 16 | ≥512 | 0.13 |
| ZH124 | 64 | 16 | 0.25 | 1 | 0.06 | 0.5 | 2 | 0.25 |
| ZH125 | 128 | 32 | ≥512 | 32 | 0.13 | 16 | 8 | 0.13 |
| ZH126 | 64 | 32 | ≥512 | 32 | 0.13 | 16 | 1 | 0.25 |
| ZH127 | 64 | 32 | ≥512 | 32 | 0.13 | 16 | 1 | 0.25 |
| ZH128 | 128 | 64 | ≥512 | 1 | 0.13 | 0.5 | 2 | 4 |
| ZH129 | 2 | 8 | 0.25 | 2 | 0.06 | 1 | 1 | 0.25 |
| ZH130 | 128 | 32 | 256 | 1 | 0.03 | 0.5 | 1 | 4 |
| ZH131 | 4 | 32 | 1 | 1 | 0.06 | 0.5 | 1 | 0.25 |
| ZH132 | 8 | 32 | 0.5 | 1 | 0.06 | 0.5 | 1 | 0.25 |
| ZH133 | 8 | 32 | 0.5 | 1 | 0.06 | 0.5 | 1 | 0.25 |
| ZH134 | 8 | 32 | 0.5 | 1 | 0.06 | 0.25 | 0.5 | 0.25 |
| ZH135 | 8 | 32 | 0.5 | 1 | 0.13 | 0.5 | 1 | 0.25 |
| ZH136 | 128 | 16 | 512 | 256 | 0.13 | 0.5 | 1 | 0.5 |
| ZH137 | 4 | 32 | 0.25 | 1 | 0.25 | 0.13 | 1 | 0.13 |
| ZH138 | 2 | 8 | 1 | 2 | 0.13 | 0.5 | 2 | 0.13 |
| ZH139 | 64 | 16 | 0.25 | 1 | 0.06 | 0.25 | 1 | 4 |
| ZH140 | 64 | 16 | 0.25 | 4 | 0.06 | 1 | 2 | 0.25 |
| ZH141 | 4 | 32 | 0.25 | 1 | 0.13 | 1 | 1 | 0.25 |
| ZH142 | 128 | 64 | 512 | 1 | 0.06 | 1 | 1 | 4 |
| ZH143 | 64 | 16 | 512 | 32 | 0.13 | 1 | 1 | 0.25 |
| ZH144 | 64 | 32 | 0.25 | 1 | 0.13 | 2 | 1 | 0.25 |
| ZH145 | 128 | 32 | 128 | 1 | 0.13 | 1 | 1 | 4 |
| ZH146 | 128 | 64 | ≥512 | 64 | 0.25 | 16 | 8 | 0.25 |
| ZH147 | 64 | 4 | 0.25 | 64 | 0.06 | 0.03 | 1 | 0.13 |
| ZH148 | 128 | 32 | ≥512 | 64 | 0.13 | 0.5 | 8 | 0.13 |
| ZH149 | 32 | 8 | ≥512 | 32 | 0.06 | 0.25 | 1 | 0.13 |
| ZH150 | 64 | 4 | 256 | 16 | 0.13 | 0.25 | 1 | 0.13 |
| ZH151 | 256 | 16 | ≥512 | 128 | 0.06 | 0.25 | 4 | 0.25 |
| ZH152 | 512 | 64 | ≥512 | 512 | 0.06 | 1 | 1 | 1 |
| ZH153 | 128 | 64 | 0.5 | 64 | 0.06 | 32 | 2 | 0.25 |
| ZH154 | 64 | 4 | 512 | 128 | 0.13 | 0.5 | ≥512 | 0.25 |
| ZH155 | 256 | 16 | 128 | 128 | 0.06 | 32 | 1 | 0.25 |
| ZH156 | 64 | 8 | 0.25 | 64 | 0.03 | 0.25 | 0.5 | 0.25 |
| ZH157 | 64 | 8 | 1 | 32 | 0.06 | 0.25 | 0.5 | 0.13 |
| ZH158 | 64 | 32 | 256 | 32 | 0.25 | 2 | 2 | 0.5 |
| ZH159 | 128 | 32 | 512 | 32 | 0.13 | 0.5 | 1 | 0.5 |
| ZH160 | 64 | 4 | ≥512 | 16 | 0.06 | 0.13 | 1 | 0.13 |
| ZH161 | 64 | 8 | 1 | 32 | 0.06 | 0.25 | 1 | 0.13 |
| ZH162 | 64 | 4 | 64 | 32 | 0.13 | 0.13 | 2 | 0.13 |
| ZH163 | 64 | 8 | 512 | 64 | 0.13 | 0.13 | ≥512 | 0.13 |
| ZH164 | 128 | 16 | 1 | 32 | 0.13 | 1 | 0.5 | 0.25 |
| ZH165 | ≥512 | 32 | ≥512 | 32 | 0.25 | 16 | 0.5 | 2 |
| ZH166 | 4 | 16 | 0.25 | 0.06 | 0.03 | 0.25 | 0.5 | 0.13 |
| ZH167 | 64 | 8 | 0.25 | 1 | 0.06 | 1 | 1 | 0.13 |
| ZH168 | 2 | 8 | 0.25 | 0.03 | 0.03 | 0.13 | 1 | 0.13 |
| ZH169 | 256 | 8 | 0.5 | 1 | 0.13 | 0.5 | 1 | 0.25 |
| ZH170 | 2 | 32 | 0.5 | 1 | 0.13 | 1 | 1 | 0.5 |
| ZH171 | 4 | 16 | 0.25 | 1 | 0.13 | 0.5 | 1 | 0.13 |
| ZH172 | 4 | 32 | 0.5 | 1 | 0.13 | 1 | 1 | 0.5 |
| ZH173 | 4 | 16 | 0.25 | 1 | 0.13 | 0.5 | 0.5 | 1 |
| ZH174 | 64 | 8 | 1 | 1 | 0.13 | 1 | 1 | 0.5 |
| ZH175 | 2 | 16 | 512 | 1 | 0.13 | 1 | 1 | 0.25 |
| ZH176 | 128 | 2 | ≥512 | 0.06 | 0.13 | 0.5 | 1 | 0.25 |
| ZH177 | 128 | 32 | 256 | 1 | 0.25 | 1 | 1 | 4 |
| ZH178 | 4 | 16 | 0.5 | 1 | 0.13 | 1 | 1 | 0.5 |
| ZH179 | 128 | 64 | ≥512 | 2 | 0.25 | 1 | 1 | 0.25 |
| ZH180 | 256 | 128 | ≥512 | 2 | 0.13 | 0.5 | 1 | 0.5 |
| ZH181 | 512 | 64 | 512 | 64 | 0.06 | 1 | 1 | 1 |
| ZH182 | 256 | 16 | ≥512 | 16 | 0.06 | 0.5 | 1 | 0.13 |
| ZH183 | 128 | 16 | 1 | 64 | 0.06 | 0.06 | 2 | 1 |
| ZH184 | 4 | 16 | 512 | 8 | 0.06 | 0.06 | 2 | 4 |
| ZH185 | 4 | 16 | 512 | 8 | 0.03 | 0.06 | 0.5 | 4 |
| ZH186 | 128 | 64 | 512 | 32 | 0.06 | 0.06 | 1 | 16 |
| ZH187 | 128 | 16 | 1 | 128 | 0.13 | 0.25 | 512 | 1 |
| ZH188 | 64 | 8 | 512 | 16 | 0.06 | 0.13 | 1 | 0.25 |
| ZH189 | 128 | 16 | 512 | 1 | 0.06 | 0.13 | 512 | 1 |
| ZH190 | 64 | 32 | 512 | 0.25 | 0.06 | 0.03 | 512 | 8 |

Notes: FFC, florfenicol; DOX, doxycycline; TIG, tigecycline; CEF, ceftiofur; IPM, imipenem; EN, enrofloxacin; AMI, amikacin; COL, colistin.
