# Supplementary material for: The flexible cotransfer of plasmids drives the dissemination of tet(X4) in swine Escherichia coli
Source: Vet Res. 2026 Apr 24;57:88. doi: 10.1186/s13567-026-01744-8 (PMC13214465; doi:10.1186/s13567-026-01744-8)
Supplement: Supplementary file 2 — Additional file 2. Genomic features of completely assembled chromosomes and plasmids obtained in this study. [file 13567_2026_1744_MOESM2_ESM.docx]

Additional file 2 Genomic features of completely assembled chromosomes and plasmids obtained in this study.

| Assembly Methods | Isolates | Chromosome or plasmid | Size(bp) | Circle | Plasmid types | BioSample number | Accession number |
| --- | --- | --- | --- | --- | --- | --- | --- |
| Unicycler | ZH142 | Chromosome (Contig_1) | 4,618,319 | Y |  | SAMN47582670 | NZ_CM125651 |
|  |  | pZH142-1 (Contig_2) | 201,203 | Y | IncHI1A/IncHIB(R27)/  IncFIA(HI1) |  | NZ_JBMIPL030000002 |
|  |  | pZH142-2 (Contig_3) | 143,847 | Y | IncFIC/IncR |  | NZ_JBMIPL030000003 |
|  |  |  |  |  |  |  |  |
|  | T142A | Chromosome (Contig_1) | 4,633,100 | Y |  | SAMN50554454 | NZ_CM125433 |
|  |  | pT142A (Contig_2) | 345,888 | Y | IncHI1A/IncHIB(R27)/  /IncFIA(HI1)/IncFIC/IncR |  | NZ_JBQGUW020000002 |
|  |  |  |  |  |  |  |  |
|  | ZH177 | Chromosome (Contig_1) | 4,910,626 | Y |  | SAMN47582672 | NZ_CM125652 |
|  |  | pZH177-1 (Contig_2) | 254,204 | Y | IncHI2/IncHI2A |  | NZ_JBMIPJ040000002 |
|  |  | pZH177-2 (Contig_3) | 99,263 | Y | p0111 |  | NZ_JBMIPJ040000003 |
|  |  | pZH177-3 (Contig_4) | 62,492 | Y | IncI2 |  | NZ_JBMIPJ040000004 |
|  |  | pZH177-4 (Contig_5) | 46,712 | Y | IncX1 |  | NZ_JBMIPJ040000005 |
|  |  | pZH177-5 (Contig_6) | 4,065 | Y |  |  | NZ_JBMIPJ040000006 |
|  |  | pZH177-6 (Contig_7) | 1,623 | Y |  |  | NZ_JBMIPJ040000007 |
|  |  |  |  |  |  |  |  |
|  | T177A | Chromosome (Contig_1) | 4633,121 | Y | chromosome | SAMN50554709 | NZ_CM125447 |
|  |  | pT177A-1 (Contig_2) | 265,142 | Y | IncHI2/IncHI2A |  | NZ_JBQIBA020000002 |
|  |  | pT177A-2 (Contig_3) | 232,447 | Y | IncHI2/IncHI2A |  | NZ_JBQIBA020000003 |
|  |  | pT177A-3 (Contig_4) | 46,712 | Y | IncX1 |  | NZ_JBQIBA020000004 |

Notes: Y denotes circular DNA.
