# Supplementary material for: The flexible cotransfer of plasmids drives the dissemination of tet(X4) in swine Escherichia coli
Source: Vet Res. 2026 Apr 24;57:88. doi: 10.1186/s13567-026-01744-8 (PMC13214465; doi:10.1186/s13567-026-01744-8)
Supplement: Supplementary file 3 — Additional file 3. Genome assembly characteristics of 24 tigecycline-resistant Escherichia coli isolates sequenced using Illumina technology. [file 13567_2026_1744_MOESM3_ESM.docx]

Additional file 3 Genome assembly characteristics of 24 tigecycline-resistant *Escherichia coli* isolates sequenced using Illumina technology.

| Assembly Methods | Isolates | Genome size (bp) | Contigs | Circle | BioSample number | Accession number |
| --- | --- | --- | --- | --- | --- | --- |
| Illumina | ZH28 | 4,809,003 | 128 | N | SAMN47582648 | NZ_JBMIQG010000000 |
|  | ZH38 | 4,743,336 | 98 | N | SAMN47582649 | NZ_JBMIQF010000000 |
|  | ZH39 | 4,965,708 | 154 | N | SAMN47582650 | NZ_JBMIQE010000000 |
|  | ZH43 | 4,849,816 | 120 | N | SAMN47582651 | NZ_JBMIQD010000000 |
|  | ZH65 | 5,088,546 | 710 | N | SAMN47582654 | NZ_JBMIQA010000000 |
|  | ZH108 | 4,972,735 | 197 | N | SAMN47582656 | NZ_JBMNYW000000000 |
|  | ZH109 | 4,938,145 | 168 | N | SAMN47582657 | NZ_JBMIPY010000000 |
|  | ZH110 | 4,852,900 | 113 | N | SAMN47582658 | NZ_JBMIPX010000000 |
|  | ZH111 | 4,955,984 | 178 | N | SAMN47582659 | NZ_JBMIPW010000000 |
|  | ZH113 | 4,888,175 | 114 | N | SAMN47582660 | NZ_JBMIPV010000000 |
|  | ZH114 | 4,928,482 | 132 | N | SAMN47582661 | NZ_JBMIPU010000000 |
|  | ZH115 | 4,919,714 | 174 | N | SAMN47582662 | NZ_JBMIPT010000000 |
|  | ZH116 | 4,930,337 | 147 | N | SAMN47582663 | NZ_JBMIPS010000000 |
|  | ZH117 | 5,359,072 | 243 | N | SAMN47582664 | NZ_JBMIPR010000000 |
|  | ZH120 | 5,101,100 | 103 | N | SAMN47582665 | NZ_JBMIPQ010000000 |
|  | ZH121 | 5,324,511 | 194 | N | SAMN47582666 | NZ_JBMIPP010000000 |
|  | ZH128 | 4,786,618 | 170 | N | SAMN47582667 | NZ_JBMIPO010000000 |
|  | ZH130 | 4,745,672 | 145 | N | SAMN47582668 | NZ_JBMIPN010000000 |
|  | ZH139 | 5,310,062 | 194 | N | SAMN47582669 | NZ_JBMIPM010000000 |
|  | ZH145 | 4,745,578 | 143 | N | SAMN47582671 | NZ_JBMIPK010000000 |
|  | ZH184 | 4,896,071 | 155 | N | SAMN47582673 | NZ_JBMIPI010000000 |
|  | ZH185 | 4,905,361 | 164 | N | SAMN47582674 | NZ_JBMIPH010000000 |
|  | ZH186 | 5,140,948 | 1386 | N | SAMN47582675 | JBMIPG010000000 |
|  | ZH190 | 4988778 | 158 | N | SAMN47582676 | NZ_JBMIPF010000000 |

Notes: N stands for linear (unclosed) DNA.
